# A qualitative exploration of mental health service user and carer perspectives on safety issues in UK mental health services

**DOI:** 10.1111/hex.13025

**Published:** 2020-02-11

**Authors:** Kathryn Berzins, John Baker, Gemma Louch, Abigail Albutt

**Affiliations:** ^1^ School of Healthcare University of Leeds Leeds UK; ^2^ NIHR Yorkshire and Humber Patient Safety Translational Research Centre Bradford Royal Infirmary Bradford Institute for Health Research Bradford UK

**Keywords:** caregivers, health services research, mental health services, patient safety

## Abstract

**Background:**

Service user and carer perspectives on safety issues in mental health services are not well known and may be important in preventing and reducing harm. The development of the Yorkshire Contributory Factors Framework—Mental Health (YCFF‐MH) provides a broad structure within which to explore these perspectives.

**Objective:**

To explore what service users of mental health services and their carers consider to be safety issues.

**Design, setting and participants:**

Qualitative interviews with 13 service users and 7 carers in the UK. Participants were asked about their experiences and perceptions of safety within mental health services. Perceived safety issues were identified using framework analysis, guided by the YCFF‐MH.

**Results:**

Service users and carers identified a broad range of safety issues. These were categorized under ‘safety culture’ and included psychological concepts of safety and raising concerns; ‘social environment’ involved threatened violence and sexual abuse; ‘individual service user and staff factors’ dominated by not being listened to; ‘management of staff and staffing levels’ resulting in poor continuity of care; and ‘service process’ typified by difficulty accessing services during a crisis. Several examples of ‘active failures’ were also described.

**Discussion and conclusions:**

Safety issues appear broader than those recorded and reported by health services and inspectorates. Many safety issues have also been identified in other care settings supporting the notion that there are overlaps between service users and carers’ perspectives of safety in mental health services and those of users in other settings. Areas for further research are suggested.

## 1 BACKGROUND

Patients can be harmed while receiving health care,^1^ and evidence suggests that between 3% and 36% of admissions to hospital result in an adverse event (eg a medication error) and up to half of these events are thought to be preventable.^2^ The majority of patient safety research has focussed on general hospital settings but has begun to include community and primary care.^3^, ^4^, ^5^ However, it is widely acknowledged that patient safety in a mental health context has received less research attention.^6^, ^7^

Over the last decade, evidence suggests that systems can be designed in general hospitals that collect feedback from patients on the safety of their care.^8^, ^9^ This research has shown that patients are willing and able to provide information about the safety of their care. This can help to identify issues not necessarily recognized by services or regulators and has the potential to inform interventions to improve safety and prevent harms and adverse events.^10^, ^11^, ^12^ Research has also explored the potential for patient involvement in patient safety within primary care^13^ and mental health services.^14^, ^15^ Given that improving safety is a priority in the delivery of mental health services, including the views of service users and their family and carers about the safety of their care is paramount.^15^

Mental health services following regulatory scrutiny by the Care Quality Commission (CQC) often need to improve the safety of care. In the UK in 2017, the CQC considered over a third of mental health services deficient in terms of safety.^16^ Of particular concern were sexual safety^17^ and the use of restrictive practices such as restraint and seclusion.^18^ Mental health services span both hospital and community settings and have significant differences to general care; for example, staff‐completed incident reports are dominated by violence and self‐harm.^19^ Harms associated with mental health services might not necessarily be caused by treatment error but by iatrogenic harms caused by medication side‐effects, the use of restraint, seclusion, forced medication or even diagnoses leading to exclusion from services.^20^ Service user and carer perceptions of the safety of mental health services have not been widely reported. Exploratory survey research collecting service user perceptions of service safety reported a broad range of issues,^21^ some echoed by service user feedback about not feeling listened to and experiencing difficulties accessing crisis support.^22^, ^23^

### 1.1 Theoretical framework

**Figure 1:**
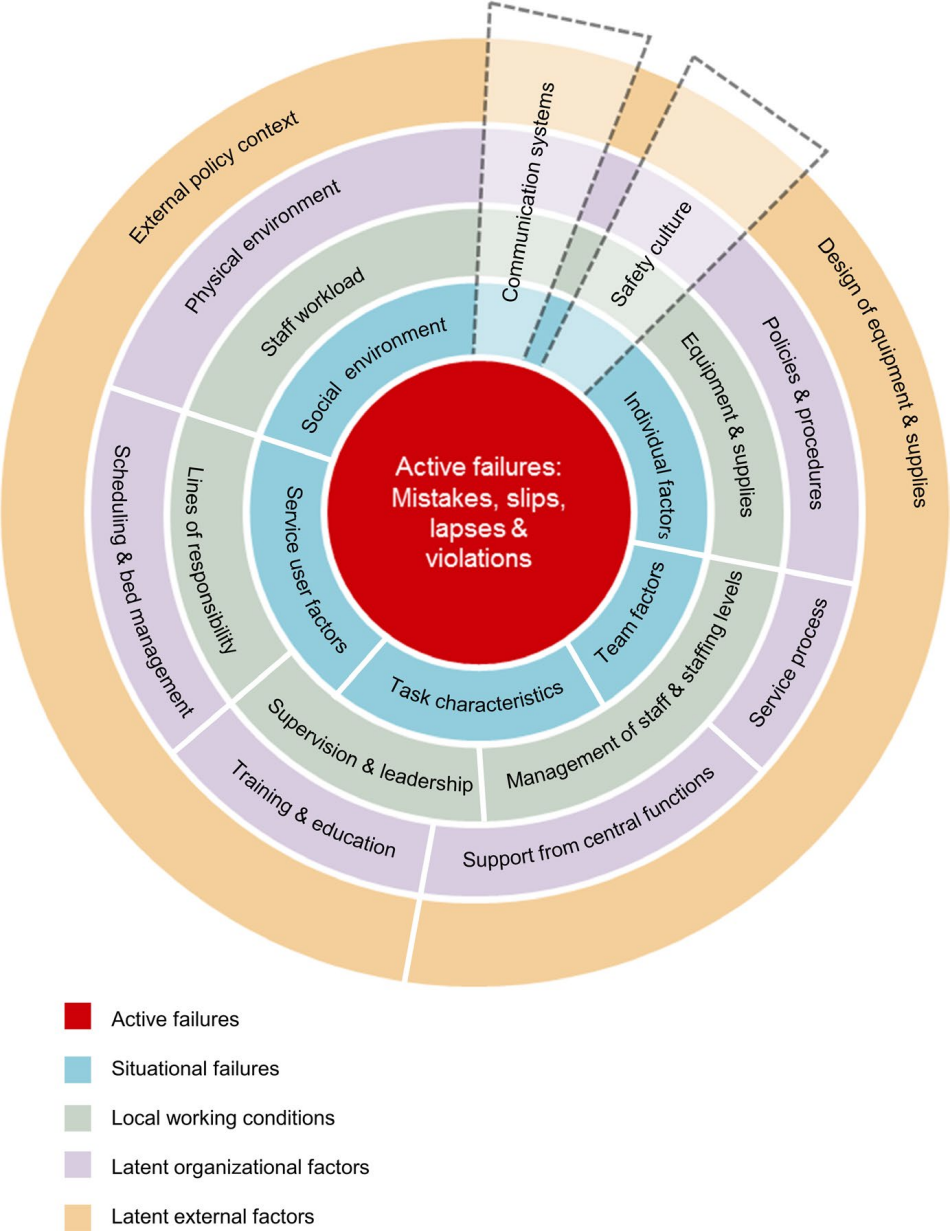
Yorkshire Contributory Factors Framework—Mental Health

The Yorkshire Contributory Factors Framework (YCFF) was systematically developed to account for factors contributing to safety incidents in general hospital care and was subsequently amended for mental health services.^21^, ^24^ The resulting Yorkshire Contributory Factors Framework—Mental Health (YCFF‐MH) consists of 20 factors (eg external policy context, physical environment, management of staff and staffing levels and individual service user and staff factors) organized into five hierarchical levels (latent external factors, latent organizational factors, local working conditions, situational factors and active failures), plus two cross‐cutting factors (communication systems and safety culture)^21^ (See Figure 1). Active failures include mistakes, slips, lapses and violations.^25^ The YCFF‐MH is intended for use to support the development of interventions to promote safety within mental health services.

The aim of this study was to explore service user and carer experiences of safety issues across mental health services using the YCFF‐MH as a theoretical basis in order to inform the development of interventions to improve the safety of mental health services.

## 2 METHODS

This study followed on from a survey study which resulted in the adapted YCFF‐MH.^21^ Given the limitations of a survey approach, we devised a qualitative study to generate a richer picture of the key safety issues and further contextualize the adapted framework. A qualitative study permitted exploration of the topic of safety. We sought to conduct semi‐structured interviews with a convenience sample of UK mental health service users and carers, recruited via social media (reported elsewhere^21^). Social media enabled recruitment of those with a breadth of experiences across many different mental health services and organizations. Service users, carers and health professionals aged over 18 with experience of contact with UK mental health services in the past two years were invited to take part in one telephone interview from their homes to discuss safety issues in services. Interviewees were assumed to be alone during the interview although as they could not be seen this may not have been the case. Due to the rich data generated, we present solely the service and carer findings in this paper. Consent forms and information sheets were emailed to people who expressed an interest. The aim of the study was reiterated prior to the interview, and verbal consent was taken and audio‐recorded. Interviewees received a £10 shopping voucher as a token of appreciation for taking part. Forty‐seven people expressed an interest to take part, 24 responded to invitation with 20 interviews conducted (13 service users, 7 carers—see Table 1). It is not known why people did not respond to the invitation. Interviews lasted between 42 and 134 minutes with a mean length of 64 minutes.

Ethical approval (reference: 12/LO/1588) was obtained from the University of [redacted] School of Healthcare Ethics Committee.

**Table 1:** Demographic characteristics

	Service users	Carers
Sex		
Female	9	5
Male	4	2
Age		
18‐25	0	—
26‐35	2	—
36‐45	3	—
46‐55	4	3
56‐65	4	4
Person cared for		
Child	‐	6
Spouse	‐	1
Experienced community care	2	‐
Experienced inpatient care	11	‐

### 2.1 Data collection

A broad interview guide was developed informed by an earlier study on the same topic.^21^ Additions were made to the guide based on early interviews, for example the issue of service user articulacy was raised by one interviewee and subsequently was raised with other interviewees. Interviews were carried out by KB, a female, PhD, academic researcher with 20 years experience researching mental health care. KB had no prior relationship with interviewees although might have interacted with their tweets on social media. Interviewees might similarly know of KB via social media. Interviewees were informed of the reasons for carrying out the research being that the team at the university were seeking to collect information about how service users and carers perceive safety issues, with the aim of developing future interventions in this area.

Interviews began with asking the interviewee to describe their background contact with services and then a broad question about what safety meant to them; if they had felt unsafe as a result of contact with services; and finally if they had raised concerns about their safety. Similar to other research exploring lay perceptions of safety,^3^ interviewees were able to define safety themselves in line with similar research.^3^ This initial discussion always led to rich narratives of experiences with services; as such, the interviewer only used the guide as a prompt if the subjects on it had not spontaneously arisen. Subjects discussed were often sensitive and distressing and external sources of support were identified on the information sheet. The interviewer ensured interviews were carried out in a supportive manner with explicit acknowledgement of the sensitive nature of the discussion and the potential for distress. Interviews were audio‐recorded with the permission of the interviewee, anonymized and transcribed verbatim. Field notes were taken.

### 2.2 Data analysis

Initial data analysis was concurrent with data collection. Interview data were analysed using framework analysis^26^ which is recognized as a useful approach when multiple researchers are working on a project and for managing large data sets which aim to generate a descriptive overview.^27^ We used a combined deductive and inductive approach. Deductive in so far as the exploration of how the data mapped to the YCFF‐MH framework, but also inductive as additional codes and sub‐codes were generated for data which did not ‘fit’ within the framework, these data were coded openly. The inductive approach allowed us to further understand a particular contributory factor in the context of mental health services at a much deeper level. For example, within the 'safety culture' coding we were able to tease out more specific coding around psychological and physical safety and raising concerns.

**Figure 2:**
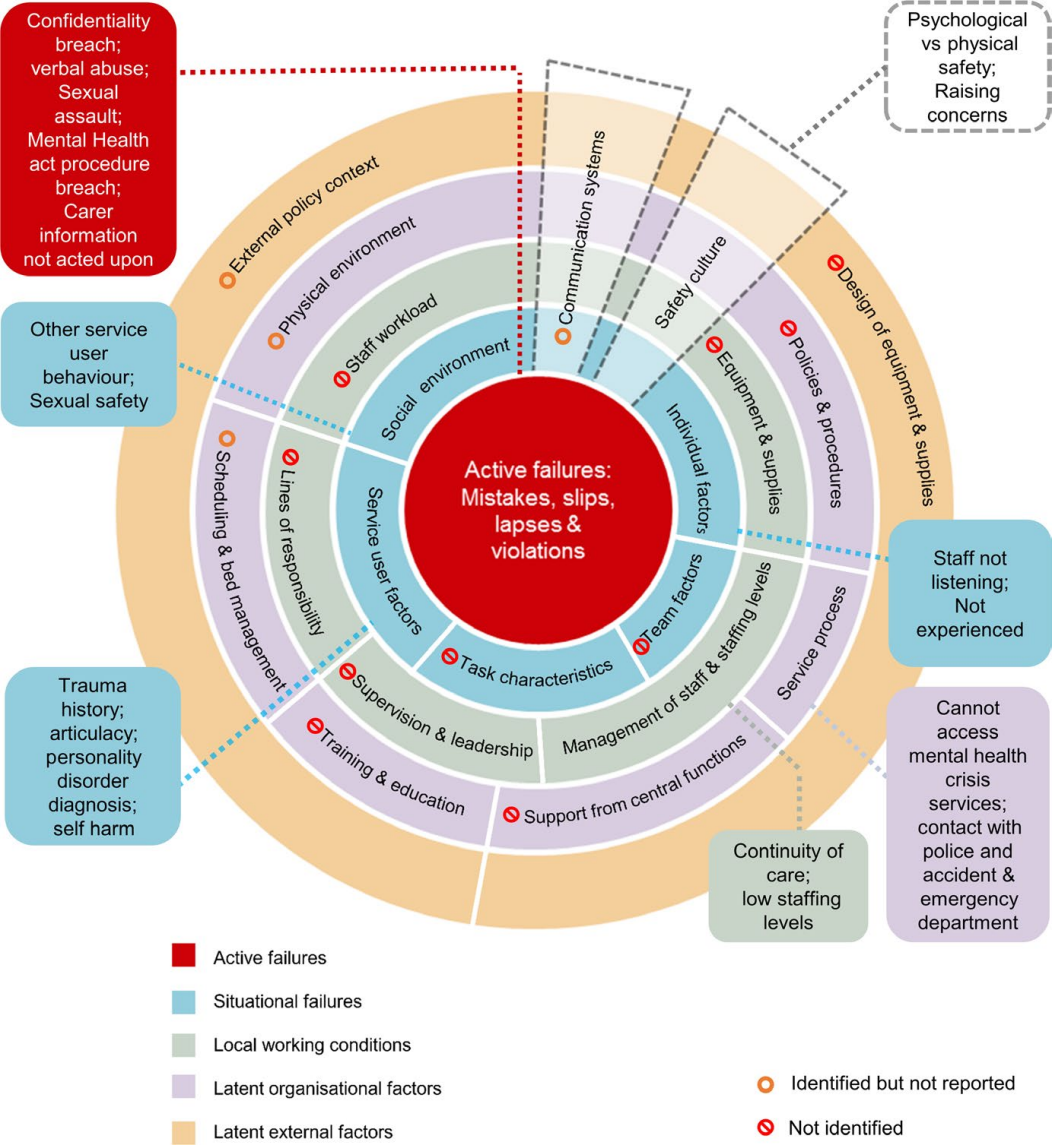
Yorkshire Contributory Factors framework—Mental Health showing factors and subthemes most frequently identified in service user and carer interviews

The analysis was supported by NVivo.^28^ Authors KB, GL and AA familiarized themselves with the transcripts prior to coding, and data could be coded onto more than one YCFF‐MH factor. Summaries of main points of each transcript were shared and discussed amongst the team before detailed coding was carried out on all transcripts by KB, with 20% of transcripts also coded by GL and AA. Any discrepancies were resolved through discussion. Data coded to the most populated factors were further assigned to subcategories to give more understanding and context (see Figure 2). Data were explored in‐depth by the three researchers during intense analysis meetings^29^; at this point, it was decided to combine the carer and service user data as analysis showed there was consensus amongst most of the issues discussed. During analysis, although we experienced ‘code saturation’ with no new codes being added, we did not consider ‘meaning saturation’ to have occurred due to the broad nature of the questioning and the diversity of participants experience.^30^ All the researchers were experienced academic researchers educated to PhD level, KB (female) and JB (male) research mental health services and GL and AA (both female) research patient safety.

### 2.3 Patient involvement in the design and conduct of the study

The focus for this research arose from general social media discussions involving authors JB and KB, who participate in social media debate with a broad range of people in their networks, including (ex)service users, family members, carers and professionals in a range of roles about improving and understanding key safety issues in mental health services. Participants were invited to consent to contact for this study as part of a larger survey; this is detailed elsewhere.^21^For the purpose of member checking, a draft of the findings was sent to interviewees to give them the opportunity to comment. Several interviewees responded to say that the findings represented their views accurately. Some interviewees provided further comments that extended beyond the scope of this paper but were useful in terms of future research.

## 3 FINDINGS

Data about safety issues related to 13 of the 20 YCFF‐MH factors. The subthemes of most frequently coded YCFF‐MH factors are shown in Figure 2. In the following sections, we provide a descriptive account of the nature of these most prominent factors and illustrative excerpts. Other factors did have some coding, for example communication systems, but are not reported.

### 3.1 Safety culture

The definition of positive safety culture within the YCFF is one where organizational values, beliefs and practices support the management of safety and learning from error.^24^ Learning from error is affected by how comfortable service users and carers feel about raising safety concerns. This section presents data which were coded according to the original YCFF definition.

#### 3.1.1 Psychological vs physical safety

Safety was conceptualized differently between service users and carers. Carers were primarily concerned with services ensuring physical safety, to prevent self‐harm and suicide. Service users described safety in services from two distinct perspectivesː physical safety (including seeking help with managing self‐harm or protection from other service users) and psychological safety (experiences within services leading to fear and distress). The two were intertwined as treatment intending to prevent physical harm, that is prevent self‐harm (particularly restraint) often caused psychological harm. The safety culture of mental health services was perceived as solely focused on avoiding physical harm at the expense of psychological harm:

How I'm feeling psychologically or mentally really isn't important as long as I'm not dead, as long as I get discharged alive, it doesn't matter what's happened to me along the way…there isn't that understanding, I don't think, of psychological safety in services, which is really odd considering it's a psychological‐based illness. Female service user ID13

One service user linked this prioritization of physical safety to professional accountability that led to paternalism:

…the problem is that staff… take on this role of risk managers where they’re seen as the ones that have to control and manage the situation which… leaves this sort of paternalistic attitude… narrows the focus of risk assessment to those things for which staff will be held accountable for, rather than the issues and concerns that service users have. Male service user ID12

Those who had experienced inpatient treatment had experienced coercion, often but not always, through having been detained under the Mental Health Act. One description of a prolonged face‐down (prone) restraint highlights how physical safety was the primary focus. The restraint might prevent further self‐harm but also caused further psychological harm.

I was restrained prone (face‐down) for almost two hours… because I'd tried to hang myself…There was no checking I was physically okay, I was just immediately prone restrained… six people restraining me … supposedly for my safety. Female service user ID13

Many interviewees had histories of abuse, some originating in mental health service; as such coercive practices were re‐traumatizing and led to fear of being re‐admitted to hospital:

…I'm scared of hospital now… I can't access out of hours care because the only out of hours care is assessment at the hospital. Female service user ID13

#### 3.1.2 Raising concerns

Interviewees frequently talked about the difficulty of raising concerns about care and safety, especially when experiencing serious enough mental health problems to be detained on a ward:

If you are ill enough to be detained, you do not have the mental energy to start formulating complaints and pursuing a complaints procedure. Female service user ID11

Carers described wanting to advocate for relatives but felt hampered by confidentiality:

I thought, well how do I make a complaint? Because people were saying to me “well you need to see his notes, you need to see his care plan”, I said “I can't, confidentiality, they don't show me anything, they won't read anything out to me… they won't tell me anything, so how can I make a complaint… how can I?” Female carer ID04

When a concern had been raised, there was often no satisfactory response:

I complained on the ward at the time… it was very defensive. I never heard anything about it, it didn’t get followed up, as far as I heard. Male carer ID05

Others felt there might be repercussions as a result of raising a concern:

My advice would be don’t complain because it will make things worse, and actually unless it's actively really harming you, you're better off to just not mention it, you're better off to just suffer it. Female service user ID13

### 3.2 Active failures

Active failures include mistakes, lapses and violations. Several active failures were described although they had few common themes. Examples included sending a confidential letter to a former address of a service user who had left there due to domestic abuse; warnings from carers with regard to antecedents to absconding and self‐harm being ignored; a Mental Health Act detention not been completed following procedure; and home visits not occurring as arranged. Experiences of violations ranged from ward staff speaking to service users in a disrespectful or taunting manner to sexual assaults:

Son had asked him something, this guy, and the guy came back at him very aggressively while I was there, and I challenged that at the time… I said, he's a patient, you know… he's not well, and I complained on the ward. Male carer ID05

…they were laughing at my distress…the female nurse, said to me, if I were you I would shut your mouth or you’ll get six months, not 28 days. Female service user ID10

Two service users described having been sexually abused by staff. These assaults had resulted in significant trauma and fear about contact with mental health services. They had disclosed these experiences to services yet did not feel that they were taken into consideration, particularly when coercive practices were used.

…I was put on a CTO to have forced medication, I tried to say to them, I really don't want to have to have an injection because it feels like a violation, it feels like assault… And they just refused. It's like your body doesn't belong to you either, which, of course, given what's happened, was awful. Female service user ID13

### 3.3 Situational factors

Situational factors include those relating to individual staff and service users, as well as the social environment on a ward.

#### 3.3.1 Social environment

Interviewees described frightening experiences caused by the behaviour of other service users resulting in an intimidating atmosphere on wards. The lack of activities was seen as a contributory factor:

…there’s definite links between having nothing to do and situations kicking off in various ways… people are just milling about with nothing to do except squabble with each other. Female service user ID11

One service user was assaulted by another service user and unable to find any staff. She had phoned the ward office using her mobile and had then been removed from the ward rather than her abuser:

I was sent to another ward which was actually not good for me because I was then in an unfamiliar environment… it felt like I was the one that suffered. …It probably was easier to move me because I was going to be less aggressive and troublesome. Female service user ID10

One carer described her son being strangled by another service user with an electrical flex she had thought should have been identified as an obvious risk in the environment:

If it wasn’t for son yelping in that moment, and a nurse seeing it and the other patient being restrained and taken away, he said son could have been killed. Female carer ID04

Sexual safety was a significant concern with mixed‐sex environments considered particularly unsafe, especially for the female interviewees with histories of sexual abuse:

I was put on a male corridor because there were no female beds; and if they’d seen my notes they would have seen that was inappropriate. Female service user ID10

#### 3.3.2 Individual service user and staff factors

Although individual service user and staff factors are separate in the YCFF‐MH, they are reported together due to the interpersonal relationship, mediated by characteristics of service users and staff, being a key element of mental health services. Some interviewees questioned the skills and experience of professionals, particularly in inpatient settings with high agency use:

If you don’t have the skill or the… sensitivity, the experience… you don’t just need numbers of unskilled bank staff that have just a week’s induction… not having a clue about mental distress. Female service user ID11

All service user interviewees spoke of how their individual characteristics (including history of trauma and previous self‐harm, diagnoses, as well how assertive they might be) influenced how they felt staff responded to them, and by extension, how this affected their perception of their safety within services. Psychological safety was threatened because staff did not listen and dismissed concerns:

…in situations where people don’t feel that they’re being listened to things then… escalate, particularly if you’re on a ward setting. Male service user ID12

Carers described how they had been assertive and warned hospital staff about signs their relative was likely to abscond or attempt suicide, but had not been taken seriously:

…every time I saw something was wrong, I forewarned people, I documented it, I wrote to people and said this is going to happen, and then when we had repeat incidents, said this is exactly the same condition as before. Male carer ID05

When carers did feel they had been listened to it had been a positive experience:

…someone came round and asked lots and lots of questions… that was useful, to have the opportunity to actually talk, and have someone listen, both to us and to my daughter. Female carer ID02

Several interviewees spoke of how being articulate could be a hindrance and led to assumptions from staff that they could maintain their own safety:

…one of the nurses on the ward said to me: ‘You're an intelligent woman, why don't you sort yourself out?’ So, yeah, because I can articulate about how I feel then… there is this feeling that you have capacity and… can't possibly be unwell. Female service user ID09

One service user interviewee had a contested diagnosis of personality disorder which she perceived had arisen from her being seen as ‘challenging’. She felt this made it less likely she would be listened to:

…the borderline personality disorder diagnosis which, in turn, made it worse because they then dismissed and fobbed me off and… it became a vicious circle. Female service user ID09

Staff not listening was reported in relation to self‐harm; carers often felt they were not listened to and repeat self‐harming was viewed as inevitable by staff, even in a ward environment:

They don't take self‐harm seriously, she managed to self‐harm multiple times whilst she was there and annoyingly it was the same things. It’s like “Yeah she’s done this before she’s going to do it again” Female carer ID03

### 3.4 Local working conditions

Local working conditions include supervision and leadership, lines of responsibility and management of staff and staffing levels. It was the latter that was most salient to interviewees.

#### 3.4.1 Management of staff and staffing levels

People had often waited a long time to receive community mental health services and found the time‐limited nature of services threatening:

I’ve only seen her twice…she has… talked a lot about being time limited… ‘at the end of this time, you’ll be discharged, and… you can always be referred back’… if I’m not ready to be discharged in the first place, why are you discharging me? Female Service user ID08

Being able to contact a known individual and continuity of staff led to greater feelings of safety, especially as the wider context and recent history were already known. There were historic examples of good relationships with specific staff members but all of them had ended, often disrupted by the person leaving their post. Several interviewees described community professionals becoming absent and not being replaced:

…to suddenly have that CPN support stopped with no phone call to say “look, your CPN is off sick, we're not sure what's happening but we can send you a duty worker once a week”…There's not even been that. Female Service user ID07

There were similar difficulties with staff on wards:

…they talk about having a therapeutic environment and people say you need to build therapeutic relationships with your nurses, well she said: “I don't know quite how I’m meant to do that when I might only see the same nurse once every fortnight”. Female Carer ID03

Staffing levels on wards were described in almost entirely negative terms. Nursing staff were not a visible presence, with their role focused on security and containment rather than therapy:

They're sitting in the office with the door shut… it always seems to me that their primary function is security. Keeping the doors locked, stopping them getting out and making sure that they’ve got their medication. Male Carer ID05

…you don’t feel like you’re in a therapeutic environment. It just feels like you’re just in a sort of containment. Male Service user ID12

Although one carer spoke favourably about her daughter being admitted to a specialist unit because of its high staffing:

…for the first time she was on one‐to‐one nursing… for the first time people actually said ‘we can help you, there’s things that can help you’. And she wasn’t allowed to harm herself… that made such a difference to her thinking because all of a sudden people were saying ‘no you can't do that we value you… Female Carer ID03

### 3.5 Latent organizational factors

Latent organizational factors included service process: the way in which people gain access to, transition between or, are discharged from services. Participants talked almost entirely in terms of gaining access to services.

#### 3.5.1 Service process

The majority of discussion about service process related to accessing timely help in a crisis. Crises often involved service users experiencing overwhelming suicidal urges; carers described having to be constantly vigilant while caring for somebody at home:

…for a long time we kept the [our] doors locked, we had to be with my daughter 24 hours a day Female carer ID02

Services were often closed out of office hours. People had been offered emergency appointments several hours or even days later when they needed immediate help to keep them safe:

…the police were called… they spoke to the crisis team and you could hear…: “we've found her, she's got a suicide note, when can you come out and assess her, please?” And the answer was: “well, we'll try and get there within five hours”. Female service user ID07

Service users who perceived they needed home visits were instead offered advice they did not think acknowledged the severity of their risk:

…take some deep breaths and read a good book! I mean, the upshot is… that if you’re in crisis and you phone up and you get told ‘Go and have a bath’ you’re not going to feel very reassured that there’s a service there to help you. Female service user ID12

This limited availability and response from crisis services meant most interviewees had experienced contact with the police or accident and emergency (A&E) departments. Experiences with the police had been mixed, some had been positive, and others had been traumatic with service users being treated in a heavy‐handed and insensitive manner:

…the police gave a human response, they saw a person in distress and they acted as one human would to another. Female service user ID10

…if somebody's made a phonecall and said there's a violent incident happening… I can understand it being heavy‐handed… but to throw me up the wall how he did… he shouldn't have grabbed me and threw me… I was just screaming, I was absolutely hysterical. Female service user ID07

The process of being strip‐searched had been traumatic for a service user already in a crisis situation:

…they stripped me of all my clothing. So somebody who's been sexually abused in hospital is now being pinned down and having all their clothing stripped off. Female service user ID13

Carers had almost entirely positive encounters with police interventions, particularly the parents of young adults who had repeatedly disappeared and been found attempting suicide or self‐harming:

I've found them helpful… they've brought her home, having found her in dire straits somewhere. And they were always very polite, and non‐judgmental. Female carer ID01

A&E was found severely lacking in specialist mental health knowledge, often confounded by long waiting times:

…their idea of an emergency is your leg hanging off. If you've cut your wrists for the tenth time this month… in their eyes it's not a real emergency. Female service user ID06

## 4 DISCUSSION

Service users and carers reflected on their experiences with mental health services to identify and describe a broad range of factors associated with safety and harm. Carers focused on physical safety and reinforced the difficulty of knowing whether another person feels unsafe and the importance of directly asking service users about their feelings on the safety of their care. In contrast, service users emphasized the psychological dimension of safety, such as fear and distress as a result of contact with services might mediate outcomes such as self‐harm, suicide and the use of restrictive practices. Safety was threatened and harm caused by active failures, particularly lapses when elements of care were omitted; the social environment of services; interaction between service users and staff; and staffing levels and access to crisis services. Interviewees accounts of harms caused by their contact (or failure to make contact) with mental health services are resonant of previous research.^31^, ^32^ Carers are not generally discussed in these terms although they can experience extreme stress as a result of trying to provide care and access help when this becomes impossible.

There has never been a state‐level recognition of harm caused by mental health services. Commentators have advocated the use of truth and reconciliation processes as a means of addressing this deficit^33^; however, the lack of state sponsorship, engagement of professionals most in need of change and retributive justice and legal reforms make them unlikely to be successful.^34^ Nevertheless, restorative justice models have been successfully applied in health services^35^ and even if not adopted systemically, have features (eg independent facilitation, acknowledgement of harm caused, reparation) that could potentially improve safety culture.^36^

Not defining safety for participants allowed them to relate it to their experiences of feeling unsafe as a result of contact with services. There were consistencies between our findings and previous safety research about mental health^21^ and primary care services.^3^, ^4^ Some interviewees would avoid particular services, for instance a crisis service, to protect their psychological safety and because they feared a hostile or dismissive response.^3^ In other care settings, patients have the right to choose between care providers and can avoid certain individuals or services.^3^, ^4^ For mental health service users however, this autonomy often cannot be exercised due to lack of treatment providers and threat of compulsory treatment, thus making it even more important that if autonomy is overridden, safety should be assured. The trauma of experiencing restrictive practices was graphically described by participants. These practices have been found to persist, despite being legally determined as a last resort.^16^ The barriers faced by service users and carers to raising concerns, having them taken seriously and responded to, echoed previous research^14^ and continue to be a thread running through health‐care service failures.^23^, ^37^

Some of the active failures described by interviewees may not have been recorded by staff on incident reporting systems. Procedural errors, assault by other patients and communication failures might be recorded by staff; however, experiences of verbal abuse from staff would require a complaint to be made, and this has been shown to be difficult.^14^ Being spoken rudely has been found to cause psychological harm to people in primary care.^4^ The incidents described in this data went beyond this and caused significant distress. Intermittent exposures of sadistic abuse committed by staff in private hospitals have shown that even in the presence of favourable CQC ratings, a severely abusive environment can be hidden from view.^38^, ^39^ The importance of listening to reports of violations from service users and their carers and relatives cannot be emphasized enough.

Safety issues in the ward social environment have been described in previous research,^21^ and the threat from other service users, including sexual assault, was the focus of a recent CQC report.^17^ Despite NHS guidance intended to eliminate same‐sex wards, there are still wards that do not fully comply.^16^ Our study reinforced these issues, although interviewees described feeling vulnerable in both single‐ and mixed‐sex environments; a single‐sex environment might reduce the likelihood of sexual assault from another service user but offers little protection from other kinds of abuse.

Interactions between service users and professionals influenced feelings of safety for all interviewees, particularly not being listened to, having wishes overridden and histories disregarded. Carers expressed frustration at not being listened to, especially about potential early warning signs that were likely to be antecedents to incidents, something frequently reported in the literature.^23^, ^40^ Service users often felt they were not listened to, which has been reported elsewhere,^22^ but not often considered within the context of safety in mental health services. Being listened to was found to be important in primary care safety research with negative consequences for both physical and mental health.^3^, ^4^, ^41^ Relationships between service users and professionals have been explored previously and primarily viewed as aspects of care quality rather than safety.^42^ It may be that the already permeable boundary between quality and safety^3^ becomes even more so once factors beyond active failures are included.

Relationships between service users and professionals were further limited by inadequate staffing levels and lack of continuity with key personnel. A similar lack of continuity was perceived by service users in primary care service safety research.^3^, ^4^, ^41^ The time‐limited nature of some services made service users feel support was only temporary and was particularly problematic when they had waited a long time. Evidence suggests more assertive patients gain access to services in primary care,^3^, ^4^ but in mental health services, assertiveness can hinder access to services with participants being seen as difficult, although carers had found being assertive had got them access in a crisis.

Accessing help in a crisis had been universally inadequate, distressing and occasionally life‐threatening for all participants. Carers spoke of their desperation when trying to prevent a seriously ill relative from harming themselves; service users spoke of their distress after encounters with non‐mental health services such as A&E and the police (although carers often spoke positively about the police). Indeed, inadequate access to crisis services is a long‐standing issue in mental health‐care services^22^, ^43^ and these findings echo those previously reported.^22^ Access also features in primary care safety research with delays leading to exacerbation of physical health problems and psychological harm.^3^, ^4^, ^41^ Recent CQC evidence gathered about access to services concluded that people have difficulty in accessing the service that is best equipped to meet their needs; one example, echoed in this study, is crisis services with limited opening hours.^16^

All these issues have been reported previously but not always within the context of safety. The CQC identifies three subthemes under their heading of safety: physical environment, staffing numbers and medication. They do not consider access to services in a crisis, psychological safety, the ability to raise concerns and use of restrictive practices to relate to safety, whereas these findings demonstrate that service users and carers clearly do.

The use of the YCFF‐MH was a key strength of the study, providing a theoretical foundation encompassing a breadth of contributory factors that included all issues raised by participants. Interviewees discussed a range of contributory factors, spread across the different levels of the framework from proximal to distal, which emphasizes the potential for improvements focused around these factors to impact on safety across whole organizations.

There was a considerable degree of overlap between the issues identified in this data and those previously identified in primary care service users’ perceptions of safety.^4^ These parallels reinforce that safety in mental health services should not be treated as a separate domain but, as has been previously advocated, as an integrated part of the discipline of patient safety.^6^

### 4.1 Recommendations for practice

Services should explore the use of the YCFF‐MH in collecting and reporting quality and safety data.

Service user and carer feedback should be proactively sought, and it should be made easier for service users to raise concerns and make complaints.

Mental health services must acknowledge when they have caused harm to service users and carers; lessons can be learned from restorative justice.

### 4.2 Limitations

This was a small‐scale, exploratory study although the findings overlap with those reported from primary care. Our codes reached saturation but we did not consider the study to have achieved ‘meaning saturation’, that is more data, particularly from carers would enrich some factors that did not have many codes associated with them.^25^ Interviewees were recruited from an online survey about safety issues, and this may mean that those who had negative experiences were more likely to participate. However, people who have not had these experiences may have less to contribute.^43^ There are many people in contact with mental health services who would not have been included in this research; using social media to publicize the study limited recruitment to users of such platforms. Different response may have emerged by recruiting directly from NHS mental health services or via alternative recruitment mechanisms which did not rely on social media.

### 4.3 Implications for future research

This study highlights the following key factors from the service user and carer perspective that are particularly important in terms of safety: safety culture; social environment; individual service user and staff factors; management of staff and staffing levels; service process, and active failures. Future research should aim to develop interventions to improve safety focused across these factors. The relationship between YCFF‐MH factors warrants further investigation, as well as exploration from the staff perspective. Its likely activity at one level can affect activity at other levels; for example management of staff and staffing levels might affect social environment. The YCFF‐MH factors not spontaneously mentioned by interviewees warrant further research; service users and carers might have valuable insights if directly questioned about issues such as 'training and education’ and ‘policies and procedures’. The findings presented here may inform the development of theory and evidence‐based instruments to measure safety from service user and carer perspectives as has been done in other settings.^44^

## 5 CONCLUSION

This study shows that service users and carers consider there to be a broad range of safety issues associated with mental health services. Interviewees described predominantly psychological harm caused not only by treatment but the behaviour of other service users, within the context of services that are understaffed and difficult to access. Patient safety has been defined as ‘the prevention of harm to patients’, and the discipline of patient safety has been defined as ‘the coordinated efforts to prevent harm, caused by the process of health care itself, from occurring to patients.'^30^ Our findings reinforce that in mental health services the definition could usefully be expanded to include harm caused when trying to access services and self‐harm provoked by contact with, or rejection from services. Efforts to improve safety in mental health services from policy level downwards, particularly accessing help in a crisis, should be underpinned by research evidence reporting the harms experienced by service user and carers; their concerns need to be central to ensure that the narrow general health service conception of safety does not continue to dominate.

## 6 Ethical approval

Ethical approval (reference:12/LO/1588) was obtained from the University of [redacted] School of Healthcare Ethics Committee.

## CONFLICT OF INTEREST

The authors declare they have no competing interests.

## Data Availability

Research data are not shared due to privacy or ethical restrictions.

## References

[1] World Health Organisation . World Alliance for Patient Safety Progress Report 2006–2007. Geneva: World Health Organisation; 2008 https://apps.who.int/iris/handle/10665/75169. Accessed May 31, 2019.

[2] The Health Foundation . Evidence Scan: Levels of Harm. London: The Health Foundation; 2011 https://www.health.org.uk/publications/levels-of-harm. Accessed May 31, 2019.

[3] Rhodes P , Campbell S , Sanders C . Trust, temporality and systems: how do patients understand patient safety in primary care? A qualitative study. Health Expect. 2016;19(2):253‐263. 25644998 10.1111/hex.12342 PMC5024004

[4] Hernan AL , Giles SJ , Fuller J , Johnson JK , Walker C , Dunbar JA . Patient and carer identified factors which contribute to safety incidents in primary care: a qualitative study. BMJ Qual Saf. 2015;24(9):583‐593. 10.1136/bmjqs-2015-004049 25972223

[5] Morris RL , Stocks SJ , Alam R , et al. Identifying primary care patient safety research priorities in the UK: a James Lind Alliance Priority Setting Partnership. BMJ Open. 2018;8(2):e020870. 10.1136/bmjopen-2017-020870 PMC5855454 29490970

[6] D'Lima D , Crawford MJ , Darzi A , Archer S . Patient safety and quality of care in mental health: a world of its own? BJPsych Bull. 2017;41(5):241‐243. 29018546 10.1192/pb.bp.116.055327 PMC5623880

[7] Shields MC , Stewart MT , Delaney KR . Patient safety in inpatient psychiatry: a remaining frontier for health policy. Health Aff. 2018;37(11):1853–1861. 10.1377/hlthaff.2018.0718 PMC10152928 30395512

[8] O'Hara JK , Armitage G , Reynolds C , et al. How might health services capture patient‐reported safety concerns in a hospital setting? An exploratory pilot study of three mechanisms. BMJ Qual Saf. 2017;26(1):42–53. 10.1136/bmjqs-2015-004260 26847140

[9] Ward JK , Armitage G . Can patients report patient safety incidents in a hospital setting? A systematic review. BMJ Qual Saf. 2012;21(8):685‐699. 10.1136/bmjqs-2011-000213 22562875

[10] Lawton R , Armitage G . The Role of the Patient in Clinical Safety. London: The Health Foundation; 2012 https://www.health.org.uk/publications/the-role-of-the-patient-in-clinical-safety. Accessed May 31, 2019.

[11] Armitage G , Moore S , Reynolds C , et al. Patient‐reported safety incidents as a new source of patient safety data: an exploratory comparative study in an acute hospital in England. J Health Serv Res Policy. 2017;23(1):36‐43. 29235364 10.1177/1355819617727563

[12] Lawton R , O'Hara JK , Sheard L , et al. Can patient involvement improve patient safety? A cluster randomised control trial of the Patient Reporting and Action for a Safe Environment (PRASE) intervention. BMJ Qual Saf. 2017;26(8):622‐631. 10.1136/bmjqs-2016-005570 PMC5537521 28159854

[13] Hernan AL , Kloot K , Giles SJ , et al. Investigating the feasibility of a patient feedback tool to improve safety in Australian primary care: a study protocol. BMJ Open. 2019;9(5):e027327. 10.1136/bmjopen-2018-027327 PMC6501999 31061052

[14] Berzins K , Louch G , Brown M , O’Hara JK , Baker J . Service user and carer involvement in mental health care safety: raising concerns and improving the safety of services. BMC Health Serv Res. 2018;18:644. 30119632 10.1186/s12913-018-3455-5 PMC6098618

[15] Dewa LH , Murray K , Thibaut B , et al. Identifying research priorities for patient safety in mental health: an international expert Delphi study. BMJ Open. 2018;8(3):e021361. 10.1136/bmjopen-2017-021361 PMC5855203 29502096

[16] Care Quality Commission . The State of Care in Mental Health Services 2014 to 2017. London: Care Quality Commission; 2018 http://www.cqc.org.uk/sites/default/files/20170720_stateofmh_report.pdf. Accessed May 31, 2019.

[17] Care Quality Commission . Sexual Safety of Mental Health Wards. London: Care Quality Commission; 2018 https://www.cqc.org.uk/publications/major-report/sexual-safety-mental-health-wards. Accessed May 31, 2019.

[18] Mind . Mental Health Crisis Care: Physical Restraint in Crisis. London: Mind; 2013.

[19] Berzins KM , Louch G , Albutt A , Baker J . Recorded incidents in UK acute mental health wards: A retrospective descriptive analysis. Poster presented at: Improving Patient Safety: New Perspectives, New Horizons, Leeds, UK.

[20] Cusack P , Cusack FP , McAndrew S , McKeown M , Duxbury J . An integrative review exploring the physical and psychological harm inherent in using restraint in mental health inpatient settings. Int J Ment Health Nurs. 2018;27(3):1162‐1176. 29352514 10.1111/inm.12432

[21] Berzins K , Baker J , Brown M , Lawton R . A cross sectional survey of mental health service user, carers and professionals priorities for patient safety in the UK. Health Expect. 2018;21(6):1085‐1094. 30120809 10.1111/hex.12805 PMC6250880

[22] Healthwatch England . What People Have Told Us About Mental Health. London: Healthwatch England; 2018 https://www.healthwatch.co.uk/report/2018-08-29/what-people-have-told-us-about-mental-health. Accessed May 31, 2019.

[23] Parliamentary and Health Services Ombudsman . Maintaining Momentum: Driving Improvements in Mental Healthcare. London: House of Commons; 2018.

[24] Lawton R , McEachan RR , Giles SJ , Sirriyeh R , Watt IS , Wright J . Development of an evidence‐based framework of factors contributing to patient safety incidents in hospital settings: a systematic review. BMJ Qual Saf. 2012;21(5):369‐380. 10.1136/bmjqs-2011-000443 PMC3332004 22421911

[25] Reason J . Human error: models and management. BMJ. 2000;320(7237):768‐770. 10720363 10.1136/bmj.320.7237.768 PMC1117770

[26] Ritchie J , Spencer L . Qualitative data analysis for applied policy research In: Analyzing Qualitative Data. London: Routledge; 2002:187‐208.

[27] Gale NK , Heath G , Cameron E , Rashid S , Redwood S . Using the framework method for the analysis of qualitative data in multi‐disciplinary health research. BMC Med Res Methodol. 2013;13(1):117. 24047204 10.1186/1471-2288-13-117 PMC3848812

[28] NVivo Qualitative Data Analysis Software. Version 12. QSR International Pty Ltd., 2018.

[29] Sheard L , Marsh C , O'Hara J , Armitage G , Wright J , Lawton R . The patient feedback response framework–understanding why UK hospital staff find it difficult to make improvements based on patient feedback: a qualitative study. Soc Sci Med. 2017;178:19‐27. 28189820 10.1016/j.socscimed.2017.02.005 PMC5360173

[30] Hennink MM , Kaiser BN , Marconi VC . Code saturation versus meaning saturation: how many interviews are enough? Qual Health Res. 2017;27(4):591‐608. 27670770 10.1177/1049732316665344 PMC9359070

[31] Frueh B , Knapp R , Cusack K , et al. Special section on seclusion and restraint: patients’ reports of traumatic or harmful experiences within the psychiatric setting. Psychiatric Services. 2005;56(9):1123‐1133. 16148328 10.1176/appi.ps.56.9.1123

[32] Spandler H . From psychiatric abuse to psychiatric neglect? Asylum Magazine. 2016;23(2):7‐8.

[33] Spandler H , Mckeown M . Exploring the case for truth and reconciliation in mental health services. Mental Health Rev J. 2017;22(2):83‐94.

[34] Cresswell M . Truth and reconciliation in psychiatry: a response to Spandler and McKeown. Mental Health Rev J. 2017;22(4):324‐331.

[35] Kaur M , De Boer RJ , Oates A , Rafferty J , Dekker S . Restorative Just Culture: A Study of the Practical and Economic Effects of Implementing Restorative Justice in an NHS Trust. In MATEC Web of Conferences 2019; 273:01007. EDP Sciences.

[36] Roddis M . Can Mandela's model for restorative justice work in healthcare? Health Serv J. 2014 https://www.hsj.co.uk/leadership/can-mandelas-model-for-restorative-justice-work-in-healthcare/5067903.article. Accessed May 31, 2019.

[37] Undercover hospital abuse scandal [Television boradcast]. Plomin, J. director. London: BBC; May 22, 2019.

[38] Undercover care: the abuse exposed [Television]. Chapman, M. director. London: BBC; May 31, 2011.

[39] Cree L , Brooks HL , Berzins K , Fraser C , Lovell K , Bee P . Carers' experiences of involvement in care planning: A qualitative exploration of the facilitators and barriers to engagement with mental health services. BMC Psychiatry. 2015;15:208. 26319602 10.1186/s12888-015-0590-y PMC4553006

[40] Ricci‐Cabello I , Pons‐Vigués M , Berenguera A , Pujol‐Ribera E , Slight SP , Valderas JM . Patients’ perceptions and experiences of patient safety in primary care in England. Fam Pract. 2016;33(5):535‐542. 27312563 10.1093/fampra/cmw046

[41] Coffey M , Cohen R , Faulkner A , Hannigan B , Simpson A , Barlow S . Ordinary risks and accepted fictions: how contrasting and competing priorities work in risk assessment and mental health care planning. Health Expect. 2017;20(3):471‐483. 27312732 10.1111/hex.12474 PMC5433531

[42] Mind . Listening to Experience: An Independent Inquiry into Acute and Crisis Mental Healthcare. London: Mind; 2011.

[43] Hernan AL , Walker C , Fuller J , Johnson JK , Abou Elnour A , Dunbar JA . Patients' and carers' perceptions of safety in rural general practice. Med J Aust. 2014;201:S60‐S63. 25047884 10.5694/mja14.00193

[44] Hernan AL , Giles SJ , O'Hara JK , Fuller J , Johnson JK , Dunbar JA . Developing a primary care patient measure of safety (PC PMOS): a modified Delphi process and face validity testing. BMJ Qual Saf. 2016;25:273‐280. 10.1136/bmjqs-2015-004268 26141502

